# Rim15 and the crossroads of nutrient signalling pathways in *Saccharomyces cerevisiae*

**DOI:** 10.1186/1747-1028-1-3

**Published:** 2006-04-03

**Authors:** Erwin Swinnen, Valeria Wanke, Johnny Roosen, Bart Smets, Frédérique Dubouloz, Ivo Pedruzzi, Elisabetta Cameroni, Claudio De Virgilio, Joris Winderickx

**Affiliations:** 1 Functional Biology, Katholieke Universiteit Leuven, Kasteelpark Arenberg 31, B-3001 Leuven-Heverlee, Belgium; 2 Department of Microbiology and Molecular Medicine, CMU, University of Geneva, Geneva, Switzerland

## Abstract

In recent years, the general understanding of nutrient sensing and signalling, as well as the knowledge about responses triggered by altered nutrient availability have greatly advanced. While initial studies were directed to top-down elucidation of single nutrient-induced pathways, recent investigations place the individual signalling pathways into signalling networks and pursue the identification of converging effector branches that orchestrate the dynamical responses to nutritional cues. In this review, we focus on Rim15, a protein kinase required in yeast for the proper entry into stationary phase (G_0_). Recent studies revealed that the activity of Rim15 is regulated by the interplay of at least four intercepting nutrient-responsive pathways.

## Background

All organisms require nutrients that provide both building blocks and energy supply to drive their metabolism and synthesize the necessary cellular components. Apart from their essential role in metabolism, certain nutrients are also potent regulators of sophisticated signalling pathways. These nutrient-induced signalling pathways allow for optimal nutrient consumption through adaptations of metabolism and concomitant adjustments of growth properties. Particularly in unicellular organisms, nutrient sensing and signalling events are of utmost importance as these organisms are often faced with dramatic environmental changes where periods of plentiful and abundant nutrient availability alternate with long periods of nutrient shortness. Microorganisms, be it lower eukaryotes or prokaryotes, rapidly resume proliferation when nutritional conditions turn favourable, but prepare themselves to enter into a resting phase (G_0_) under condition where essential nutrients start to become limiting [1,2]. In higher multicellular organisms, most cells experience relative environmental homeostasis, while a sub-set of specialised cell types continually monitor the level of key nutrients and produce appropriate metabolic and/or behavioural responses, typically via altered secretion of certain hormones and/or neurotransmitters. A well-known example is the regulation of human blood glucose via the insulin and glucagon hormones produced in specialised pancreatic islet cells [3,4]. Interestingly, recent evidence shows that particular nutrients can initiate cell-signalling events independently of hormonal influences also in higher eukaryotes [5,6].

**Figure 1:**
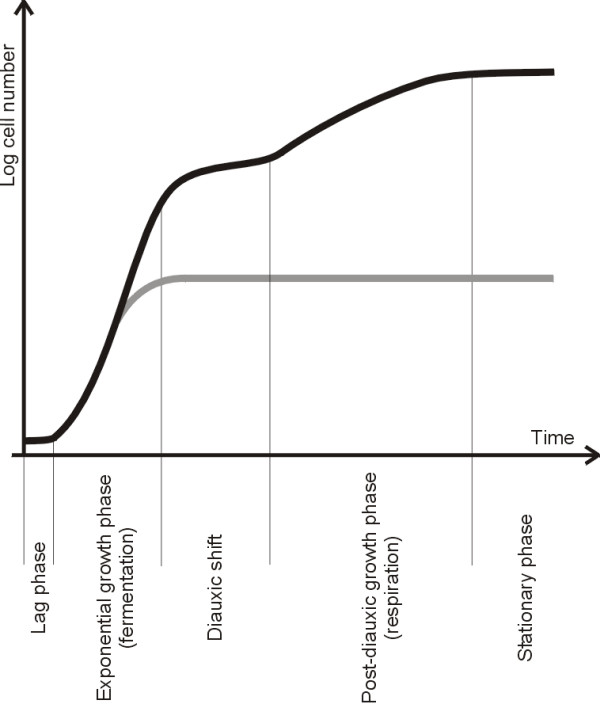
**Typical culture-density profile of a fermentative batch culture of *S. cerevisiae***. Black line: A schematic representation of the increase in cell number and cell density of a batch culture of Saccharomyces cerevisiae inoculated in rich medium containing a rapidly fermentable sugar, e.g. glucose, as a carbon source. After a short adaptive lag phase, yeast consumes the sugar during the exponential fermentative growth phase. When the sugar becomes limiting, yeast cells enter the diauxic shift and reprogram their metabolic capacity from fermentation to respiration. In the post-diauxic growth phase, the cells consume ethanol, acetate and other products of the initial fermentation as carbon source. Finally, when these carbon sources are exhausted, the cells enter a quiescent state, the stationary phase (G_0_), with the ultimate goal of surviving the starvation period. Gray line: When exponentially growing yeast cells are transferred to medium containing glucose but missing an essential nutrient such as nitrogen or phosphate, they arrest growth and enter the G_0 _state due to nutrient deprivation.

Baker's yeast *Saccharomyces cerevisiae *has proven to be an important model organism for studies related to nutrient-induced signalling events, which is underlined by the fact that many signal transduction mechanisms are highly conserved from yeast to higher eukaryotes [7]. For yeast cells, rapidly fermentable sugars are the preferred carbon and energy sources. Hence, when glucose is added to yeast cells grown on a non-fermentable carbon source, they rapidly adapt their metabolism to fermentation during a short lag-phase to ensure optimal and exclusive use of this rich carbon source. This adaptation requires different regulatory pathways such as the glucose-repression pathway and the Ras-cAMP pathway [8]. After this initial transition, the cells start to consume and ferment the sugar during the exponential phase where they display a maximal growth rate (Fig. 1). Once glucose becomes limiting, yeast cells enter a second lag-phase, known as diauxic shift during which they reset their metabolic mode from fermentation to respiration. This metabolic switch is followed by a second slow-growing phase during which ethanol, acetate and other products of the initial fermentation process are being used as carbon sources. Finally, when these carbon sources are exhausted, the cells enter a quiescent or stationary phase (G_0_). During entry into the G_0 _phase, many transcriptional and metabolic rearrangements take place in the yeast cell. Some of these (such as the induction of stress-responsive genes and the accumulation of the reserve carbohydrate trehalose) serve to acquire stress resistance and ensure optimal survival during the starvation period. Other changes such as the repression of genes involved in protein synthesis result in the controlled downregulation of growth. Interestingly, when yeast cells are starved (in the presence of glucose) for essential nutrients such as for instance nitrogen or phosphate, similar physiological adjustments can be observed than the one observed following glucose limitation. This observation indicates that several components involved in the control of typical G_0_-traits may be shared between multiple nutrient-induced signalling pathways. Moreover, certain environmental stresses (*e.g., *heat stress) engender entry of cells into a G_0_-like state, indicating that nutritional deprivation can be considered as a specific stress condition [9,10]. Consistent with this idea, nutrient and stress signalling pathways were found to converge on components like Msn2 and Msn4 (Msn2/4), a pair of partially redundant transcription factors that regulate expression of a subset of stress-responsive genes that contain stress-responsive elements (STREs) in their promoters [11-13]. In this review we focus on the protein kinase Rim15. While originally identified as a regulator of meiosis in diploid cells, recent studies demonstrated that Rim15 is essential in both diploid and haploid cells for the proper entry into G_0_. Most interestingly, Rim15 was found to integrate signals derived from several different nutrient-sensory kinases (*i.e*., PKA, TORC1, Sch9, and Pho85-Pho80) that transmit information on the availability of different nutrients.

**Figure 2:**
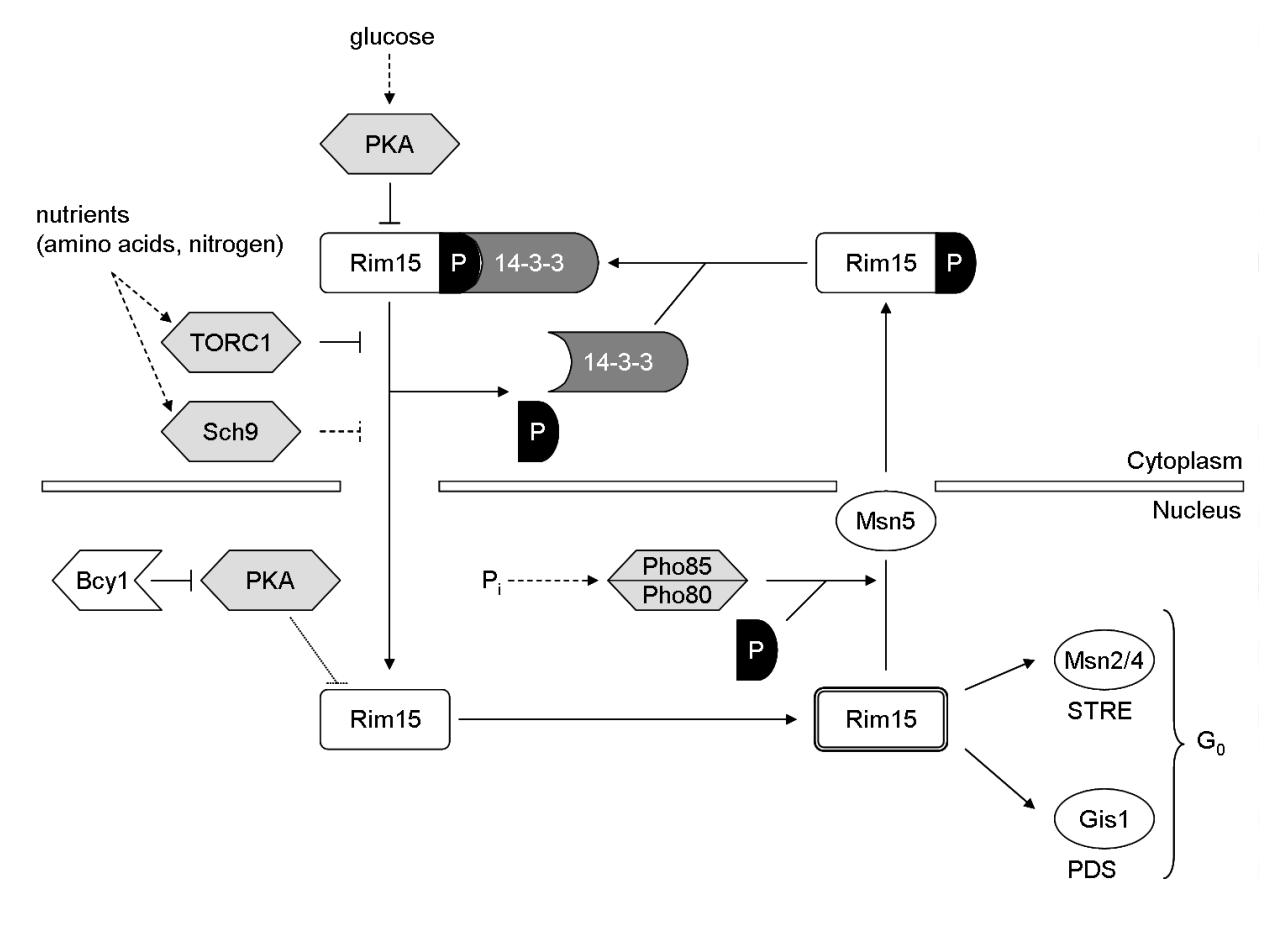
**Integration of nutrient signals by Rim15**. Rim15 activity is regulated by at least four nutrient-regulated protein kinases. Cytoplasmic Rim15, anchored through its binding to the 14-3-3 proteins, is kept inactive through PKA-mediated phosphorylation. TORC1 inactivation results in dephosphorylation of phospho-Thr1075 (indicated with P) in Rim15 and concomitant translocation of Rim15 to the nucleus where it escapes further PKA-mediated inhibition (as Bcy1 inhibits PKA activity in the nucleus). Also the Pho85-Pho80 kinase is involved in phosphorylation of Thr1075 in Rim15, thereby promoting cytoplasmic retention of nuclear exported Rim15. The mechanism by which Sch9 inhibits the nuclear localisation of Rim15 is not known. Active nuclear Rim15 regulates entry into the stationary (G_0_) phase via regulation of the Msn2,4 and Gis1 transcription factors. Msn2/4 regulate expression of stress responsive (STRE) genes while Gis1 induces transcription of post-diauxic shift (PDS) genes. Active Rim15 also initiates an autophosphorylation process (illustrated by the double line around Rim15) which stimulates its nuclear export via Msn5. See text for details.

## Identification of Rim15 as regulator of meiosis

Diploid yeast cells undergo meiosis under conditions of nitrogen depletion in the presence of a non-fermentable carbon source such as acetate [14,15]. At the onset of meiosis, early meiotic-specific genes (EMGs) are transiently expressed. Induction of these genes depends on a switch of function of the Ume6 protein. Under repressing conditions, Ume6 recruits the histone deacetylases (HDAC) Rpd3 and Sin3 to the promoters of EMGs, resulting in transcriptional repression. When meiosis is induced Rpd3 and Sin3 are transiently removed from EMG promoters and Ume6 recruits the transcriptional activator Ime1, which results in histone acetylation and transcription of EMGs such as *IME2 *[16].

Using a genetic screen, several mutations were isolated that caused reduced expression of an *ime2-lacZ *fusion gene and the corresponding genes were named *RIM *(Regulator of *IME2*) genes [17]. The *rim15/rim15 *mutant displayed a reduced ability to undergo meiosis, due to reduced expression of both *IME1 *and EMGs [17,18]. Cloning of the *RIM15 *gene identified a serine-threonine protein kinase of 1770 amino acids, which was subsequently found to promote the functional interaction between Ume6 and Ime1 by stimulating the removal of the Rpd3/Sin3 HDAC from the promoters of EMGs in response to depletion of glucose and nitrogen [16,18,19]. The expression of Rim15 is repressed in the presence of glucose medium and its role during meiosis appears to be confined in regulating the first step in meiosis, *i.e. *the transient induction of EMGs [18].

## Rim15 as downstream effector of PKA that regulates entry into G0 via the transcription factors Gis1, Msn2 and Msn4

Independently of the studies on meiosis, Rim15 was also isolated as a protein that specifically interacted with the Tps1 subunit of the trehalose synthase complex [20,21]. Detailed phenotypic analyses revealed that loss of Rim15 causes a pleiotropic phenotype in cells grown to stationary phase on rich medium; this phenotype includes defects in trehalose and glycogen accumulation, in transcriptional derepression of *HSP12*, *HSP26*, and *SSA3*, in induction of thermotolerance and starvation resistance, and in proper G_1 _arrest. As all of these traits are reminiscent of deficiencies previously ascribed to mutations that cause constitutive activation of PKA, these observations pointed to a role of Riml5 in nutrient signalling at some point in the Ras-cAMP pathway. Tests of epistasis indeed supported the notion that Rim15 may act in this pathway downstream of protein kinase A (PKA). Accordingly, while loss of Rim15 rendered cells independent of PKA activity, overproduction of Rim15 exacerbated the growth defect of strains compromised for PKA activity. Finally, biochemical analyses revealed that PKA-mediated phosphorylation of Rim15 (specifically at five PKA consensus phosphorylation sites; RRXS) strongly inhibited its kinase activity *in vitro*. Taken together, these results placed Rim15 immediately downstream and under negative control of PKA and defined a positive regulatory role of Rim15 in G_0 _entry [21].

Adaptation to nutrient starvation conditions involves critical reprogramming of overall transcription. Interestingly, a dosage-suppressor screen of the defect of *rim15*Δ mutants to induce transcription of *SSA3 *following nutrient limitation led to the identification of Gis1, which is a zinc-finger transcription factor that binds the post-diauxic shift (PDS) element in the promoter of genes that become derepressed upon glucose exhaustion at the diauxic shift [22]. The core of the PDS-element closely resembles the consensus sequence of the STRE-element, which confers transcriptional induction in response to a variety of stresses including nutrient limitation and oxidative, heat and osmotic stress. Interestingly, the control of STRE-driven gene expression by PKA is mediated by the transcription factors Msn2 and Msn4 [11-13]. Moreover, both Msn2 and Msn4 partially overlap in functions with Gis1 *in vivo *and appear to regulate transcription of a large set of genes in a cooperative manner [22-24]. In line with this finding, combination of the *gis1*Δ mutation with the *msn2Δ msn4*Δ mutations causes an apparent synthetic growth defect on non-fermentable carbon sources [24]. Important to the discussion here is that genome-wide transcriptional profiling indicated the three transcription factors, Msn2, Msn4, and Gis1, to almost entirely account for the Rim15-dependent effects on transcription during glucose limitation at the diauxic shift [23]. The mechanisms by which Rim15 controls the functions and activities of Gis1 and Msn2/4, however, are still unknown. Although direct phosphorylation of the transcription factors by Rim15 is conceivable, Rim15 may alternatively regulate the establishment of physiological context-specific interactions between these factors and the general transcription machinery [25] or activate transcription from Msn2/4- and Gis1-dependent promoters through chromatin remodeling [26,27].

## Integration of nutrient signals via Rim15

The data described above established the epistatic relation between PKA, Rim15 and the transcription factors Gis1, Msn2 and Msn4. In addition, with Rim15 being immediately downstream PKA, the data also provided a molecular link between the Ras-cAMP pathway and the regulation of meiosis because activation of PKA inhibits Rim15, thereby preventing the dissociation of Ume6 from Sin3/Rpd3 and ensuring repression of EMGs. This was further confirmed *in vivo *since Rpd3 is not bound to the EMG promoters in sporulation medium supplemented with 2% glucose in a strain that expresses the constitutively active 5-RRXS/A mutant allele of *RIM15 *[16].

Notably, enhanced synthesis of cAMP is mainly controlled by glucose, but not by any other nutrient [8]. Since yeast cells also respond to nitrogen or phosphate starvation by acquiring many of the same G_0_-characteristics as glucose-starved cells, additional mechanisms must exist that integrate the information on the availability on these other nutrients [8,28,29]. Notably, in this context, entry into meiosis requires integration of two nutritional cues (*i.e., *the absence of a fermentable carbon source and the limited supply of nitrogen), underlining the proposed existence of additional nutrient integration mechanisms [14,15].

Several studies dealing with the regulation of molecular processes associated with the availability of glucose and nitrogen implicated the TOR pathway in the nutrient-responsive signalling network of yeast. The central components of this pathway are the highly conserved protein kinases Tor1 and Tor2, which were both identified based on a screen for mutants that confer resistance to the immunosuppressant rapamycin. Tor1 and Tor2 are present in two functionally distinct multiprotein complexes, *i.e. *TORC1, which controls ribosome biogenesis, translation, protein turnover and transcription of starvation-specific genes, and TORC2, which controls actin cytoskeleton organization. Only the TORC1 complex is inhibited by the immunosuppressant rapamycin [30-32]. Cells treated with rapamycin arrest growth and induce a typical G_0 _program and therefore phenotypically resemble nutrient-starved cells. Intriguingly, mutants that lack Rim15 fail to properly enter G_0 _following rapamycin treatment as exemplified for instance by their corresponding defects both in transcriptional induction of PDS- and STRE-driven genes and in trehalose and glycogen accumulation. The underlying reason for this is that rapamycin treatment and/or inhibition of TORC1 abolishes the cytoplasmic retention and thereby favours the cytoplasmic-to-nuclear translocation of Rim15 [33]. Mechanistically, inactivation of TORC1 results in dephosphorylation of the pThr^1075 ^in a 14-3-3-binding site of Rim15 thereby causing the release of Rim15 from the cytoplasmic 14-3-3 anchor protein Bmh2 [34]. Notably, this TORC1 effect on Rim15 appears not to involve the known TORC1-effector Sit4 and it is possibly also independent of the type 2A protein phosphatases Pph21 and Pph22 [33].

Another pathway involved in signalling the presence of glucose and nitrogen to yeast cells is the so-called Fermentable Growth Medium (FGM) induced pathway [8]. The key component of this pathway is the protein kinase Sch9, an orthologue of mammalian PKB/Akt. Initially, Sch9 was proposed to act as a nutritional regulator that controls the activity of the free catalytic subunits of PKA via a cAMP-independent process [28]. Recent genetic data, however, indicated that Sch9 might converge with the Ras-cAMP pathway downstream of PKA on its effector Rim15 [24,33]. Like TORC1, Sch9 is required for cytoplasmic retention of Rim15 during exponential growth on glucose-containing medium [33]. While the epistatic relationship between TORC1 and Sch9 remains to be elucidated, it was proposed that Sch9 may be involved in phosphorylation of a second (pThr^1075^-independent) 14-3-3-binding site in Rim15, which may act in concert with the 14-3-3-binding site flanking Thr1075 to mediate tandem 14-3-3 binding [34]. Furthermore, it is worth noting that Rim15 nuclear accumulation can only be visualized using kinase-inactive variant versions of GFP-Rim15 indicating that autophosphorylation of Rim15 stimulates its proper nuclear export [34]. Such a mechanism may add an additional layer to the dynamic control of G_0 _entry by establishing a corresponding requirement for continued starvation signals to maintain sufficiently high levels of Rim15 in the nucleus.

In addition to its role in glucose- and nitrogen-dependent signalling, Rim15 also controls entry into G_0 _in response to a phosphate (Pi) starvation signal [29]. Signalling phosphate starvation in yeast is mediated by the PHO pathway, which consists of the cyclin-dependent kinase (CDK) Pho85 and its cyclin partner Pho80, the CDK inhibitor (CKI) Pho81 and the Pho4 transcription factor [35]. Under high-Pi conditions, Pho85-Pho80 phosphorylates and thereby inactivates the Pho4 transcription factor. Upon Pi starvation, Pho81 inhibits the Pho85-Pho80 kinase complex, thereby allowing activation of Pho4 and the concomitant transcriptional induction of genes involved in the acquisition, uptake and storage of phosphate [36,37]. Importantly, Pi starvation induces, in a Rim15-dependent manner, similar G_0_- traits as those described above following glucose and nitrogen starvation [29,38]. In support of these genetic studies, biochemical studies have recently revealed that Rim15 represents a *bona fide *target of the Pho85-Pho80 kinase complex both *in vitro *as well as *in vivo *[34]. Surprisingly, a previously defined minimum domain of Pho81, necessary and sufficient to regulate Pho4 action in response to Pi availability [39], is deficient in regulating Rim15 activity in response to Pi starvation [29]. Therefore, distinct domains of Pho81 have to be involved in the regulation of the two targets (*i.e*., Pho4 and Rim15) of the Pho85-Pho80 kinase complex.

Intriguingly, Pho85-Pho80 phosphorylates Thr^1075 ^within the 14-3-3 binding site of Rim15. Thus, Pho85-Pho80 stimulates Bmh2-dependent, cytoplasmic retention of Rim15 via phosphorylation of the 14-3-3 site flanking the residue Thr1075, while TORC1 likely controls the phosphorylation status of the same residue by keeping corresponding pThr^1075^-targeting phosphatases inactive. Notably, since Pho85-Pho80 is localized in the nucleus and TORC1 in the cytoplasm [40,41], both kinases are likely to act on different pools of Rim15. Taken together, based on current knowledge Rim15 integrates signals from at least four nutrient-sensitive protein kinases, namely TORC1, Sch9 and Pho85-Pho80, which control sub-cellular localization, and PKA, which controls the activity of Rim15.

An important question, which remains to be addressed, is whether each of these nutrient-sensitive kinases (*i.e., *TORC1, Sch9, Pho85-Pho80, and PKA) converges independently on Rim15, or whether they may impinge on each other at different levels. Current data suggest that Pho85-Pho80 acts independently of TORC1 and Sch9 [29,34]. Whether PKA acts independently of TORC1 and/or Sch9, however, is still a matter of debate [24,33,42,43]. Undoubtedly, understanding of the relationship between these nutrient-signalling kinases will require more detailed knowledge on both the operating feedback mechanisms in the corresponding pathways and the identification of additional critical points of convergence. One such point may be Msn2, whose nuclear exclusion/cytoplasmic retention is apparently controlled independently by both PKA and TORC1 [24,44-47]. Moreover, our current data suggest that Gis1 may actually represent another important point of convergence that integrates signals from both Sch9 and Pho85-Pho80 independently [24,29].

## Rim15 represents a new, distinct member of the PAS kinase family

**Figure 3:**
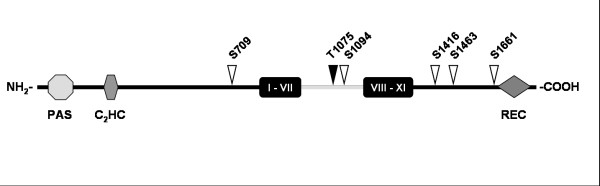
**Schematic diagram illustrating the domain architecture of the *S. cerevisiae *Rim15 protein.** All domains are drawn approximately to scale. Rim15 belongs to a group of conserved fungal proteins, which exhibit the same domain organization, including the N-terminal PAS and CCHC-type zinc finger domains, the central kinase catalytic domain (black ovals), with an insert of 188 amino acids between subdomains VII and VIII, that classifies Rim15 as a member of the conserved nuclear Dbf2-related (NDR) and large tumor suppressor (LATS) serine/threonine kinase subclasses of the protein kinase A, G, and C (AGC) class of kinases, and a C-terminal receiver (REC) domain. The PKA and the Pho85-Pho80 phosphorylation sites are indicated with open and closed arrows, respectively. The single high-stringency, putative 14-3-3 protein-binding site in Rim15 flanks amino acid T1075.

Rim15 is a distant member of the NDR family of AGC kinases, which share the presence of an insert between the protein kinase subdomains VII and VIII [18,23,48]. In *S. cerevisiae*, this insert comprises 188 amino acids and contains a binding site for the 14-3-3 protein Bmh2, which anchors Rim15 in the cytoplasm under nutrient-rich conditions [18,34]. The kinase domain of Rim15 is flanked by large N- and C-terminal extensions containing several functional domains (Fig. 3). These include an evolutionary conserved, amino-terminal PAS (Per-Arnt-Sim) domain, which classifies Rim15 as a new, distinct member of the PAS kinase family [23]. PAS domains are a small regulatory modules represented in all kingdoms of life and function as *cis*-acting regulatory domains that may respond to a wide variety of stimuli, such as changes in light, redox potential, oxygen, small ligands and overall cellular energy levels [49-53]. The identification of a PAS domain in Rim15 opens the perspective that this domain might act as a *cis*-regulatory switch that regulates Rim15 in response to additional signals besides the nutrient-induced regulation described above. An interesting candidate signal represents oxidative stress. As yeast cells traverse the diauxic shift, the rise in mitochondrial respiratory chain activity produces an increase in reactive oxygen species (ROS) [54]. Superoxide, a typical ROS, inactivates the aconitase and other [4Fe-4S] cluster enzymes and may cause loss of mitochondrial function and ultimately cell death [55,56]. In response to oxidative stress, yeast cells therefore regulate the induction of various protective mechanisms. Rim15 seems to play a crucial role in this process since *rim15*Δ mutant cells display a decreased chronological life-span when compared to wild-type strains [57]. Interestingly, Rim15 was proposed to act downstream of the Ras-cAMP-PKA and Sch9 pathways and to regulate transcriptional induction of the mitochondrial manganese superoxide dismutase-encoding *SOD2 *gene [23,57,58]. Since *rim15*Δ but not *msn2Δ msn4*Δ or *sod2*Δ mutants display a specific short-lived phenotype [57,58], Rim15 is likely to regulate longevity via yet additional mechanisms. An intriguing model posits that the PAS domain may sense oxidative stress and/or cellular redox potential and regulate Rim15 activity in response to these signals [23].

## Future and perspectives

Although several regulatory mechanisms controlling Rim15 activity have recently been elucidated, some important questions remain unanswered. These include the nature of the presumed TORC1-controlled protein phosphatase(s) that controls the phosphorylation status of Thr^1075^. Moreover, the molecular details by which Sch9 controls Rim15 subcellular localisation [33], the role of the Rim15-PAS domain for intra- or inter-molecular regulation of the Rim15 kinase activity [23], or the mechanism by which defined domains in Pho81 regulate Rim15 function [29,34], remain key issues that will have to be addressed in the near future.

A major challenge in further understanding Rim15 function lies within the identification of *bona fide *physiological targets of this kinase. Although Rim15 phosphorylates the Ume6 protein *in vitro *[19], it is still unclear whether this also occurs *in vivo*. Additional candidate target proteins may be the Sin3 and/or Rpd3 HDAC proteins [16]. Moreover, even though Msn2/4 and Gis1 were genetically identified as downstream targets of Rim15, it is possible that Rim15 may rather indirectly control the activity of these transcription factors [22-24]. Interestingly, a recent high-throughput *in vitro *kinase assay has identified several potential candidates of the Rim15 kinase [59]. Even though none of the above mentioned putative target proteins were scored in this assay, analysis of the corresponding positive hits may yield most valuable information regarding the cellular function of Rim15.

In conclusion, Rim15 plays a central role in connecting nutrient-induced signalling pathways, thereby regulating crucial processes involved in yeast proliferation, differentiation and aging. As many of these processes are highly conserved among eukaryotes, the studies in yeast may provide important clues on the molecular mechanisms that govern these processes in higher organisms as well.
